# 2-[(4-Chloro­benz­yl)carbonyl­meth­yl]benzoic acid

**DOI:** 10.1107/S160053680803451X

**Published:** 2008-10-25

**Authors:** Obaid-ur-Rahman Abid, Ghulam Qadeer, Nasim Hasan Rama, Ales Ruzicka

**Affiliations:** a Department of Chemistry, Quaid-i-Azam Univeristy, Islamabad 45320, Pakistan; b Department of General and Inorganic Chemistry, Faculty of Chemical Technology, University of Pardubice, Nam. Cs. Legii’ 565, 53210 Pardubice, Czech Republic

## Abstract

The title compound, C_16_H_13_ClO_3_, is an important inter­mediate in the conversion of isocoumarin to 3,4-dihydro­isocoumarin. The two aromatic rings are oriented at a dihedral angle of 67.18 (3)°. In the crystal structure, inter­molecular O—H⋯O hydrogen bonds link the mol­ecules into centrosymmetric dimers. There is also a C—H⋯π contact between the benzoic acid and 4-chloro­benzyl rings.

## Related literature

For a related structure, see: Abid *et al.* (2006 ▶). For general background, see: Barry (1964 ▶); Powers *et al.* (2002 ▶); Rossi *et al.* (2003 ▶); Sturtz *et al.* (2002 ▶); Thomas & Jens (1999 ▶). For bond-length data, see: Allen *et al.* (1987 ▶).
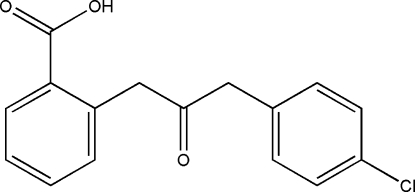


## Experimental

### 

#### Crystal data

C_16_H_13_ClO_3_
*M* *_r_* = 288.71
Monoclinic, 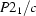

*a* = 5.5000 (4) Å
*b* = 13.2720 (6) Å
*c* = 18.8120 (7) Å
β = 94.371 (4)°
*V* = 1369.21 (13) Å^3^
*Z* = 4
Mo *K*α radiation
μ = 0.28 mm^−1^
*T* = 150 (1) K
0.29 × 0.19 × 0.16 mm

#### Data collection

Bruker–Nonius Kappa CCD area-detector diffractometer
Absorption correction: integration (Coppens, 1970 ▶) *T*_min_ = 0.936, *T*_max_ = 0.962
10076 measured reflections
3010 independent reflections
2284 reflections with *I* > 2σ(*I*)
*R*_int_ = 0.048

#### Refinement

*R*[*F* ^2^ > 2σ(*F* ^2^)] = 0.050
*wR*(*F* ^2^) = 0.118
*S* = 1.14
3010 reflections
181 parameters
H-atom parameters constrained
Δρ_max_ = 0.26 e Å^−3^
Δρ_min_ = −0.42 e Å^−3^

### 

Data collection: *COLLECT* (Hooft, 1998 ▶); cell refinement: *COLLECT* and *DENZO* (Otwinowski & Minor, 1997 ▶); data reduction: *COLLECT* and *DENZO*; program(s) used to solve structure: *SIR92* (Altomare *et al.*, 1994 ▶); program(s) used to refine structure: *SHELXL97* (Sheldrick, 2008 ▶); molecular graphics: *PLATON* (Spek, 2003 ▶); software used to prepare material for publication: *SHELXL97*.

## Supplementary Material

Crystal structure: contains datablocks I, global. DOI: 10.1107/S160053680803451X/hk2557sup1.cif

Structure factors: contains datablocks I. DOI: 10.1107/S160053680803451X/hk2557Isup2.hkl

Additional supplementary materials:  crystallographic information; 3D view; checkCIF report

## Figures and Tables

**Table 1:** Hydrogen-bond geometry (Å, °)

*D*—H⋯*A*	*D*—H	H⋯*A*	*D*⋯*A*	*D*—H⋯*A*
O2—H2⋯O1^i^	0.82	1.81	2.626 (3)	176
C16—H16⋯*Cg*1^ii^	0.93	3.35	4.079 (3)	137

Symmetry codes: (i) 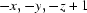; (ii) 
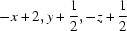
. *Cg*1 is the centroid of the C2–C7 ring.

**Fig. 1.**
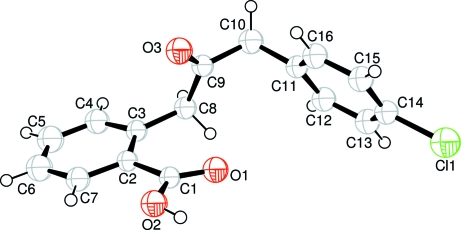
The molecular structure of the title molecule, with the atom-numbering scheme.

**Fig. 2.**
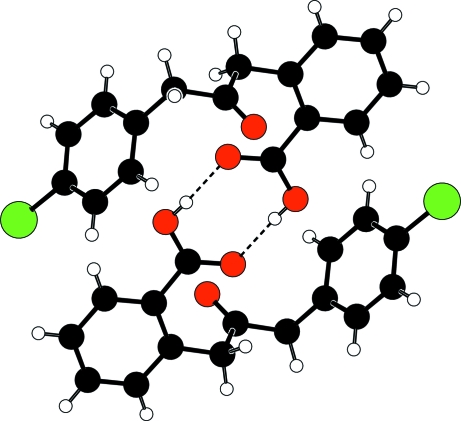
A partial packing diagram. Hydrogen bonds are shown as dashed lines.

**Fig. 3.**
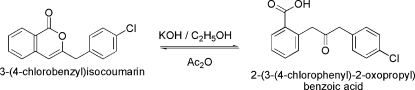
The formation of the title compound.

## supplementary crystallographic information

### Comment

The isocoumarin nucleus is an abundant structural motif in natural products (Barry, 1964). Many constituents of the steadily growing class of known isocoumarins exhibit valuable biological properties such as antifungal (Sturtz *et al.*,  2002), antitumor or cytotoxic, anti-inflammatory, anti-allergic (Rossi *et al.*,  2003) and enzyme inhibitory activity (Powers *et al.*,  2002). Naturally occurring haloisocoumarins and their halogeno-3,4-dihydroisocoumarin derivatives are very rare. However, a few examples of naturally occurring chlorine containing isocoumarins are known (Thomas & Jens, 1999). In view of the importance of this class of compounds, the title compound, an intermediate during the conversion of isocoumarin to 3,4-dihydroisocoumarin, has been synthesized, and we report herein its crystal structure.

In the title compound (Fig. 1), the bond lengths (Allen *et al.*, 1987) and angles are within normal ranges, and comparable with the corresponding values in 3-(2-chlorobenzyl)isocoumarin (Abid *et al.*,  2006). Rings A (C2-C7) and B (C11-C16) are, of course, planar and the dihedral angle between them is A/B = 67.18 (3)°. The intramolecular C-H···O hydrogen bonds (Table 1) result in the formation of nonplanar five- and six-membered rings C (O2/C1/C2/C7/H7) and D (O1/C1-C3/C8/H8B). Ring C adopts envelope conformation with C1 atom displaced by -0.108 (3) Å from the plane of the other ring atoms, while ring D has twisted conformation.

In the crystal structure, intermolecular O-H···O hydrogen bonds (Table 1) link the molecules into centrosymmetric dimers (Fig. 2), in which they may be effective in the stabilization of the structure. There also exist a C—H···π contact (Table 1) between the benzoic acid and 4-chlorobenzyl rings.

### Experimental

A solution of 3-(4-chlorobenzyl)isocoumarin (2.0 g, 7 mmol) in ethanol (50 ml) and potassium hydroxide (100 ml, 5%) were refluxed for 4 h. Ethanol was removed from the reaction mixture by distillation. Ice cold water (20 ml) was added and the reaction mixture was acidified with hydrochloric acid. It was extracted with dichloromethane (3 × 20 ml), and then dried and evaporated to yield the crude solid, which was recrystallized from methanol (yield; 85%; m.p. 414-415 K).

### Refinement

H atoms were positioned geometrically, with O-H = 0.82 Å (for OH) and C-H = 0.93 and 0.97 Å for aromatic and methylene H, respectively, and constrained to ride on their parent atoms with U_iso_(H) = 1.2U_eq_(C,O).

### Crystal data

**Table:**

C_16_H_13_ClO_3_	*F*(000) = 600
*M**_r_* = 288.71	*D*_x_ = 1.401 Mg m^−^^3^
Monoclinic, *P*2_1_/*c*	Melting point: 414(1) K
Hall symbol: -P 2ybc	Mo *K*α radiation, λ = 0.71073 Å
*a* = 5.5000 (4) Å	Cell parameters from 10141 reflections
*b* = 13.2720 (6) Å	θ = 1–27.5°
*c* = 18.8120 (7) Å	µ = 0.28 mm^−^^1^
β = 94.371 (4)°	*T* = 150 K
*V* = 1369.21 (13) Å^3^	Block, colorless
*Z* = 4	0.29 × 0.19 × 0.16 mm

### Data collection

**Table:**

Bruker–Nonius Kappa CCD area-detector diffractometer	3010 independent reflections
Radiation source: fine-focus sealed tube	2284 reflections with *I* > 2σ(*I*)
graphite	*R*_int_ = 0.048
Detector resolution: 9.091 pixels mm^-1^	θ_max_ = 27.5°, θ_min_ = 1.9°
φ and ω scans	*h* = −6→7
Absorption correction: integration (Coppens, 1970)	*k* = −17→15
*T*_min_ = 0.936, *T*_max_ = 0.962	*l* = −21→24
10076 measured reflections	

### Refinement

**Table:**

Refinement on *F*^2^	Primary atom site location: structure-invariant direct methods
Least-squares matrix: full	Secondary atom site location: difference Fourier map
*R*[*F*^2^ > 2σ(*F*^2^)] = 0.050	Hydrogen site location: inferred from neighbouring sites
*wR*(*F*^2^) = 0.118	H-atom parameters constrained
*S* = 1.14	*w* = 1/[σ^2^(*F*_o_^2^) + (0.0291*P*)^2^ + 0.9198*P*] where *P* = (*F*_o_^2^ + 2*F*_c_^2^)/3
3010 reflections	(Δ/σ)_max_ < 0.001
181 parameters	Δρ_max_ = 0.26 e Å^−^^3^
0 restraints	Δρ_min_ = −0.42 e Å^−^^3^

### Special details

**Table:**

Geometry. All e.s.d.'s (except the e.s.d. in the dihedral angle between two l.s. planes) are estimated using the full covariance matrix. The cell e.s.d.'s are taken into account individually in the estimation of e.s.d.'s in distances, angles and torsion angles; correlations between e.s.d.'s in cell parameters are only used when they are defined by crystal symmetry. An approximate (isotropic) treatment of cell e.s.d.'s is used for estimating e.s.d.'s involving l.s. planes.
Refinement. Refinement of *F*^2^ against ALL reflections. The weighted *R*-factor *wR* and goodness of fit *S* are based on *F*^2^, conventional *R*-factors *R* are based on *F*, with *F* set to zero for negative *F*^2^. The threshold expression of *F*^2^ > σ(*F*^2^) is used only for calculating *R*-factors(gt) *etc*. and is not relevant to the choice of reflections for refinement. *R*-factors based on *F*^2^ are statistically about twice as large as those based on *F*, and *R*- factors based on ALL data will be even larger.

### Fractional atomic coordinates and isotropic or equivalent isotropic displacement parameters (Å^2^)

**Table:**

	*x*	*y*	*z*	*U*_iso_*/*U*_eq_	
Cl1	0.56140 (14)	0.35295 (5)	0.41926 (4)	0.0597 (2)	
O1	0.1626 (3)	−0.05908 (11)	0.44337 (9)	0.0445 (4)	
O2	−0.1720 (3)	−0.11437 (12)	0.48932 (9)	0.0446 (4)	
H2	−0.1636	−0.0593	0.5091	0.054*	
O3	0.0425 (3)	−0.11324 (13)	0.27714 (9)	0.0487 (4)	
C1	0.0174 (4)	−0.12684 (15)	0.45249 (11)	0.0312 (4)	
C2	0.0445 (4)	−0.23057 (15)	0.42498 (10)	0.0313 (4)	
C3	0.2232 (4)	−0.25579 (16)	0.37852 (11)	0.0338 (5)	
C4	0.2425 (5)	−0.35656 (18)	0.35955 (13)	0.0470 (6)	
H4	0.3604	−0.3753	0.3292	0.056*	
C5	0.0930 (5)	−0.42938 (18)	0.38464 (15)	0.0547 (7)	
H5	0.1120	−0.4963	0.3714	0.066*	
C6	−0.0840 (5)	−0.40396 (18)	0.42911 (14)	0.0512 (6)	
H6	−0.1869	−0.4529	0.4456	0.061*	
C7	−0.1076 (4)	−0.30469 (17)	0.44895 (12)	0.0410 (5)	
H7	−0.2273	−0.2869	0.4790	0.049*	
C8	0.3863 (4)	−0.18048 (17)	0.34637 (12)	0.0378 (5)	
H8A	0.5133	−0.2165	0.3238	0.045*	
H8B	0.4648	−0.1402	0.3845	0.045*	
C9	0.2595 (4)	−0.11086 (16)	0.29233 (11)	0.0354 (5)	
C10	0.4211 (4)	−0.03623 (19)	0.25726 (13)	0.0458 (6)	
H10A	0.5798	−0.0665	0.2528	0.055*	
H10B	0.3504	−0.0211	0.2096	0.055*	
C11	0.4526 (4)	0.06038 (17)	0.29935 (11)	0.0363 (5)	
C12	0.6641 (4)	0.07862 (19)	0.34231 (13)	0.0437 (6)	
H12	0.7855	0.0297	0.3464	0.052*	
C13	0.6968 (4)	0.16829 (19)	0.37871 (13)	0.0459 (6)	
H13	0.8396	0.1800	0.4072	0.055*	
C14	0.5170 (4)	0.23985 (16)	0.37280 (11)	0.0391 (5)	
C15	0.3062 (4)	0.22462 (18)	0.33044 (13)	0.0432 (5)	
H15	0.1860	0.2740	0.3262	0.052*	
C16	0.2761 (4)	0.13451 (18)	0.29391 (13)	0.0430 (5)	
H16	0.1334	0.1235	0.2652	0.052*	

### Atomic displacement parameters (Å^2^)

**Table:**

	*U*^11^	*U*^22^	*U*^33^	*U*^12^	*U*^13^	*U*^23^
Cl1	0.0813 (5)	0.0436 (4)	0.0545 (4)	−0.0193 (3)	0.0070 (3)	−0.0008 (3)
O1	0.0533 (10)	0.0334 (8)	0.0491 (10)	−0.0093 (7)	0.0181 (8)	−0.0123 (7)
O2	0.0431 (9)	0.0377 (9)	0.0549 (10)	−0.0034 (7)	0.0157 (8)	−0.0169 (7)
O3	0.0400 (9)	0.0498 (10)	0.0547 (10)	0.0010 (8)	−0.0064 (7)	0.0059 (8)
C1	0.0330 (11)	0.0308 (10)	0.0297 (10)	0.0027 (9)	0.0012 (8)	−0.0025 (8)
C2	0.0362 (11)	0.0269 (10)	0.0300 (10)	0.0030 (9)	−0.0028 (8)	−0.0027 (8)
C3	0.0365 (11)	0.0342 (11)	0.0297 (10)	0.0071 (9)	−0.0045 (8)	−0.0057 (9)
C4	0.0560 (15)	0.0397 (13)	0.0446 (13)	0.0147 (11)	−0.0015 (11)	−0.0126 (11)
C5	0.0716 (18)	0.0266 (11)	0.0633 (17)	0.0077 (12)	−0.0123 (14)	−0.0102 (11)
C6	0.0652 (17)	0.0299 (12)	0.0567 (16)	−0.0076 (12)	−0.0075 (13)	0.0017 (11)
C7	0.0470 (13)	0.0343 (12)	0.0409 (12)	−0.0043 (10)	−0.0009 (10)	−0.0001 (9)
C8	0.0331 (11)	0.0430 (12)	0.0375 (11)	0.0079 (10)	0.0042 (9)	−0.0073 (10)
C9	0.0386 (12)	0.0345 (11)	0.0334 (11)	0.0041 (9)	0.0046 (9)	−0.0085 (9)
C10	0.0484 (14)	0.0492 (14)	0.0414 (13)	0.0000 (11)	0.0139 (10)	−0.0019 (11)
C11	0.0353 (11)	0.0410 (12)	0.0337 (11)	−0.0017 (9)	0.0096 (9)	0.0053 (9)
C12	0.0346 (12)	0.0512 (14)	0.0449 (13)	0.0089 (10)	0.0005 (10)	0.0104 (11)
C13	0.0394 (13)	0.0566 (15)	0.0404 (12)	−0.0076 (11)	−0.0067 (10)	0.0060 (11)
C14	0.0453 (13)	0.0351 (11)	0.0371 (12)	−0.0104 (10)	0.0052 (10)	0.0075 (9)
C15	0.0388 (12)	0.0393 (12)	0.0515 (14)	0.0029 (10)	0.0025 (10)	0.0069 (11)
C16	0.0324 (11)	0.0496 (14)	0.0463 (13)	−0.0025 (10)	−0.0024 (9)	0.0028 (11)

### Geometric parameters (Å, °)

**Table:**

Cl1—C14	1.745 (2)	C8—C9	1.505 (3)
O2—H2	0.8200	C8—H8A	0.9700
O3—C9	1.207 (3)	C8—H8B	0.9700
C1—O1	1.223 (2)	C9—C10	1.515 (3)
C1—O2	1.305 (2)	C10—H10A	0.9700
C1—C2	1.482 (3)	C10—H10B	0.9701
C3—C4	1.390 (3)	C11—C10	1.510 (3)
C3—C2	1.404 (3)	C11—C12	1.386 (3)
C4—H4	0.9300	C11—C16	1.381 (3)
C5—C4	1.376 (4)	C12—H12	0.9300
C5—C6	1.373 (4)	C13—C12	1.378 (4)
C5—H5	0.9300	C13—C14	1.369 (3)
C6—H6	0.9299	C13—H13	0.9300
C7—C2	1.389 (3)	C15—C14	1.371 (3)
C7—C6	1.378 (3)	C15—C16	1.383 (3)
C7—H7	0.9299	C15—H15	0.9299
C8—C3	1.501 (3)	C16—H16	0.9299
			
C1—O2—H2	109.6	O3—C9—C8	122.9 (2)
O1—C1—O2	122.49 (19)	O3—C9—C10	121.1 (2)
O1—C1—C2	123.35 (18)	C8—C9—C10	116.01 (19)
O2—C1—C2	114.13 (18)	C11—C10—C9	111.99 (18)
C7—C2—C3	120.05 (19)	C11—C10—H10A	109.2
C7—C2—C1	117.74 (19)	C9—C10—H10A	109.2
C3—C2—C1	122.16 (18)	C11—C10—H10B	109.3
C4—C3—C2	117.4 (2)	C9—C10—H10B	109.3
C4—C3—C8	118.5 (2)	H10A—C10—H10B	107.9
C2—C3—C8	124.08 (18)	C16—C11—C12	118.2 (2)
C5—C4—C3	121.9 (2)	C16—C11—C10	120.9 (2)
C5—C4—H4	119.1	C12—C11—C10	120.8 (2)
C3—C4—H4	119.1	C13—C12—C11	120.8 (2)
C6—C5—C4	120.4 (2)	C13—C12—H12	119.7
C6—C5—H5	119.8	C11—C12—H12	119.5
C4—C5—H5	119.7	C14—C13—C12	119.5 (2)
C5—C6—C7	119.1 (2)	C14—C13—H13	120.2
C5—C6—H6	120.6	C12—C13—H13	120.2
C7—C6—H6	120.3	C13—C14—C15	121.2 (2)
C6—C7—C2	121.2 (2)	C13—C14—Cl1	118.91 (18)
C6—C7—H7	119.5	C15—C14—Cl1	119.86 (18)
C2—C7—H7	119.3	C14—C15—C16	118.7 (2)
C3—C8—C9	114.86 (18)	C14—C15—H15	120.7
C3—C8—H8A	108.7	C16—C15—H15	120.6
C9—C8—H8A	108.6	C11—C16—C15	121.5 (2)
C3—C8—H8B	108.4	C11—C16—H16	119.3
C9—C8—H8B	108.6	C15—C16—H16	119.2
H8A—C8—H8B	107.5		

### Hydrogen-bond geometry (Å, °)

**Table:**

*D*—H···*A*	*D*—H	H···*A*	*D*···*A*	*D*—H···*A*
O2—H2···O1^i^	0.82	1.81	2.626 (3)	176
C7—H7···O2	0.93	2.32	2.669 (3)	102
C8—H8B···O1	0.97	2.33	2.790 (3)	108
C16—H16···Cg1^ii^	0.93	3.35	4.079 (3)	137

Symmetry codes: (i) −*x*, −*y*, −*z*+1; (ii) −*x*+2, *y*+1/2, −*z*+1/2.
